# The Morphology and Performance of Poly(Vinyl Chloride) Containing Melamine Schiff Bases against Ultraviolet Light

**DOI:** 10.3390/molecules24040803

**Published:** 2019-02-22

**Authors:** Gamal A. El-Hiti, Mohammad Hayal Alotaibi, Ahmed A. Ahmed, Basheer A. Hamad, Dina S. Ahmed, Ahmed Ahmed, Hassan Hashim, Emad Yousif

**Affiliations:** 1 Department of Optometry, College of Applied Medical Sciences, King Saud University, P.O. Box 10219, Riyadh 11433, Saudi Arabia; 2 National Center for Petrochemicals Technology, King Abdulaziz City for Science and Technology, P.O. Box 6086, Riyadh 11442, Saudi Arabia; mhhalotaibi@kacst.edu.sa or malotaibi1@gmail.com; 3 Department of Chemistry, College of Science, Al-Nahrain University, Baghdad 64021, Iraq; ahmedahmedalazawi@gmail.com (A.A.A); basheerali400@gmail.com (B.A.H.); 4 Department of Medical Instrumentation Engineering, Al-Mansour University College, Baghdad 64021, Iraq; dinasaadi86@gmail.com; 5 Polymer Research Unit, College of Science, Al-Mustansiriyah University, Baghdad 10052, Iraq; drahmed625@gmail.com; 6 Department of Physics, College of Science, Al-Nahrain University, Baghdad 64021, Iraq; hassan.albattat@gmail.com

**Keywords:** poly(vinyl chloride), melamine, Schiff bases, ultraviolet irradiation, carbonyl group index, photostabilization, weight loss

## Abstract

Five Schiff bases derived from melamine have been used as efficient additives to reduce the process of photodegradation of poly(vinyl chloride) films. The performance of Schiff bases has been investigated using various techniques. Poly(vinyl chloride) films containing Schiff bases were irradiated with ultraviolet light and any changes in their infrared spectra, weight, and the viscosity of their average molecular weight were investigated. In addition, the surface morphology of the films was inspected using a light microscope, atomic force microscopy, and a scanning electron micrograph. The additives enhanced the films resistance against irradiation and the polymeric surface was much smoother in the presence of the Schiff bases compared with the blank film. Schiff bases containing an ortho-hydroxyl group on the aryl rings showed the greatest photostabilization effect, which may possibly have been due to the direct absorption of ultraviolet light. This phenomenon seems to involve the transfer of a proton as well as several intersystem crossing processes.

## 1. Introduction

Plastics have unique chemical and physical properties and low production costs. They are produced in huge quantities and are involved in various applications [1]. However, the chemical industry has come under severe pressure of late to overcome the problems created due to the use of plastics, such as the amount of waste generated, the nature of such waste, and their detrimental effect on the environment. In addition, one of the main concerns is how the properties of plastics may be improved for long term use [2]. Poly(vinyl chloride) (PVC) is a common and versatile thermoplastic that is utilized in daily applications ranging from food packaging to medical devices.

PVC is cheap and has relatively high chemical and biological resistance. However, PVC has poor thermal and mechanical stability. In addition, most of the common forms of synthetized PVC suffer from a particular dehydrochlorination process when exposed to ultraviolet (UV) light for long periods, particularly at high temperatures [3]. Such processes lead to the formation of carbon–carbon double bonds within the polymeric chains due to the elimination of hydrogen chloride (HCl), and may also cause discoloration, deformation, cracking, and loss of mechanical properties [4]. Moreover, the degradation of PVC in landfill sites is slow under natural conditions [5]. Therefore, the PVC waste has to be either incinerated, or picked and recycled, at the various landfill sites where it ends up [6]. The incineration of PVC produces toxic by-products that not only pollute the environment but also harm soil and groundwater [7]. Therefore, the incorporation of thermal and UV stabilizers within the polymeric matrix could be a solution to enhance the properties of PVC [8,9].

The PVC additives should be compatible with the polymeric chains and should not alter the color of the product. Moreover, they should be stable, non-volatile, and cheap to produce [10]. The most common PVC additives are metal complexes [11,12,13,14], aromatic rich compounds [15,16,17,18], Schiff bases [19,20,21,22], nanomaterials, flame retardants, and pigments [23,24,25]. However, stabilizers containing heavy metals are not good for the long-term protection of PVC since the metals have a tendency to leach out, thereby causing environmental and health problems. Titanium dioxide can be used as a PVC additive since it acts as an effective UV light absorber [26,27]. However, titanium dioxide is also known as a photocatalyst and, therefore, accelerates PVC surface weathering and degradation [28]. Accordingly, there is still room to develop new PVC additives that are efficient, easy to produce, highly aromatic, and act as both thermal and UV stabilizers. Melamine is aromatic, odorless, non-toxic, cheap, and highly stable. It is used in various industrial applications, such as corrosion inhibitors [29,30,31]. Therefore, we became interested in the use of melamine-based additives to reduce the effects of photodegradation of PVC. Now, we aim to report the synthesis of Schiff bases containing melamine as efficient PVC photostabilizers as part of our interest in the field of polymers [32,33,34,35,36,37,38,39].

## 2. Results and Discussion

### 2.1. Schiff Bases ***1***−***5***

**Figure 1:**
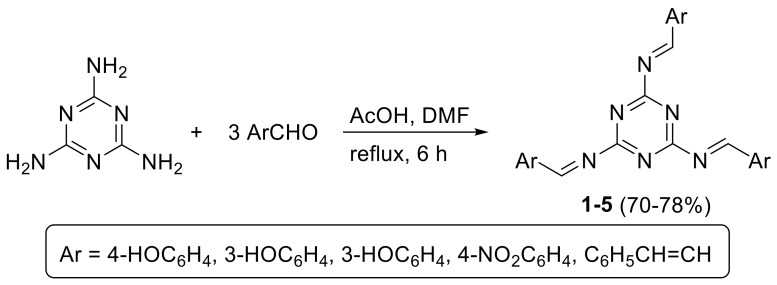
Synthesis of Schiff bases **1**–**5**.

Schiff bases **1**–**5** were synthesized based on a previously reported procedure [40] from the condensation of melamine and three molar equivalents of aromatic aldehydes—namely 2-hydroxybenzaldehyde, 3-hydroxybenzaldehyde, 4-hydroxybenzaldehyde, 4-nitrobenzaldehyde and cinnamaldehyde—in boiling dimethylformamide containing acetic acid (Figure 1). Schiff bases **1**–**5** were obtained in 70–78% yields as pale-yellow solids. The elemental analyses and some physical properties for **1**–**5** are shown in Table 1.

Fourier transform infrared (FTIR) spectra of **1**–**5** (Table 2) showed strong absorption peaks at 1632–1647 cm^–1^ region due to the vibration of azomethine bonds. The vibrations of aromatic C=C and triazine C=N bonds appeared at 1438–1465 and 1539–1543 cm^–1^ region, respectively. Proton nuclear magnetic resonance (^1^H-NMR) spectra showed characteristic singlets that resonated within the 8.48–9.29 ppm region corresponding to the azomethine protons (Table 3).

### 2.2. Energy Dispersive X-Ray Spectroscopy

**Figure 2:**
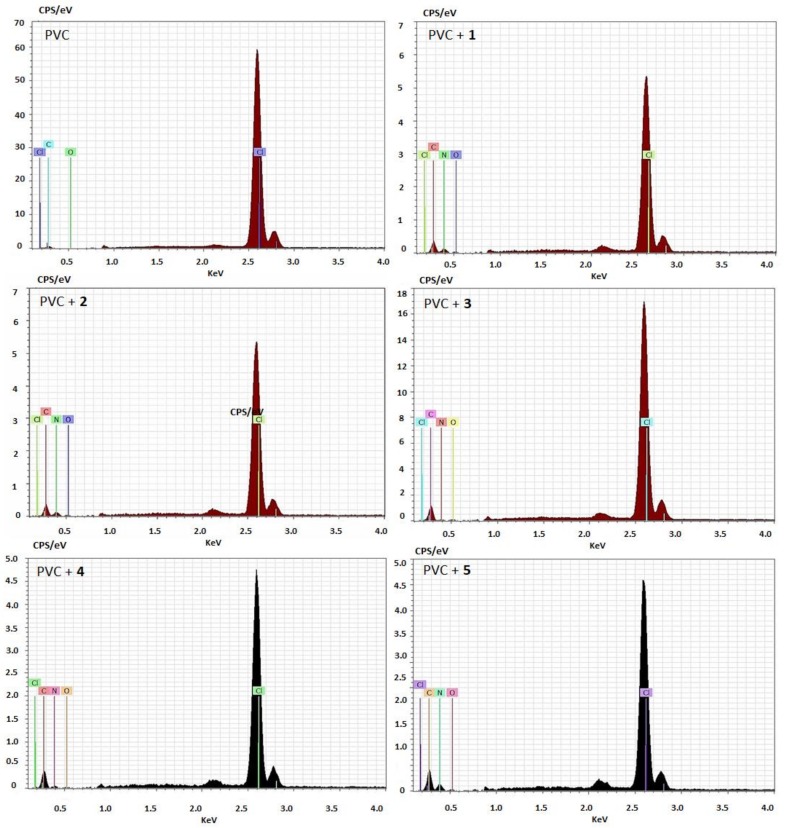
Energy dispersive X-ray (EDX) patterns of poly(vinyl chloride) (PVC).

Schiff bases **1**–**5** (0.5% by weight) were incorporated within the PVC matrix to produce blends. Following this, energy dispersive X-ray (EDX) spectroscopy was used to determine the elements within the polymeric materials. The EDX patterns for PVC/**1**–**5** blends clearly showed the appearance of a new band that corresponded with the nitrogen atoms of the additives (Figure 2).

### 2.3. FTIR Spectroscopy

**Figure 3:**
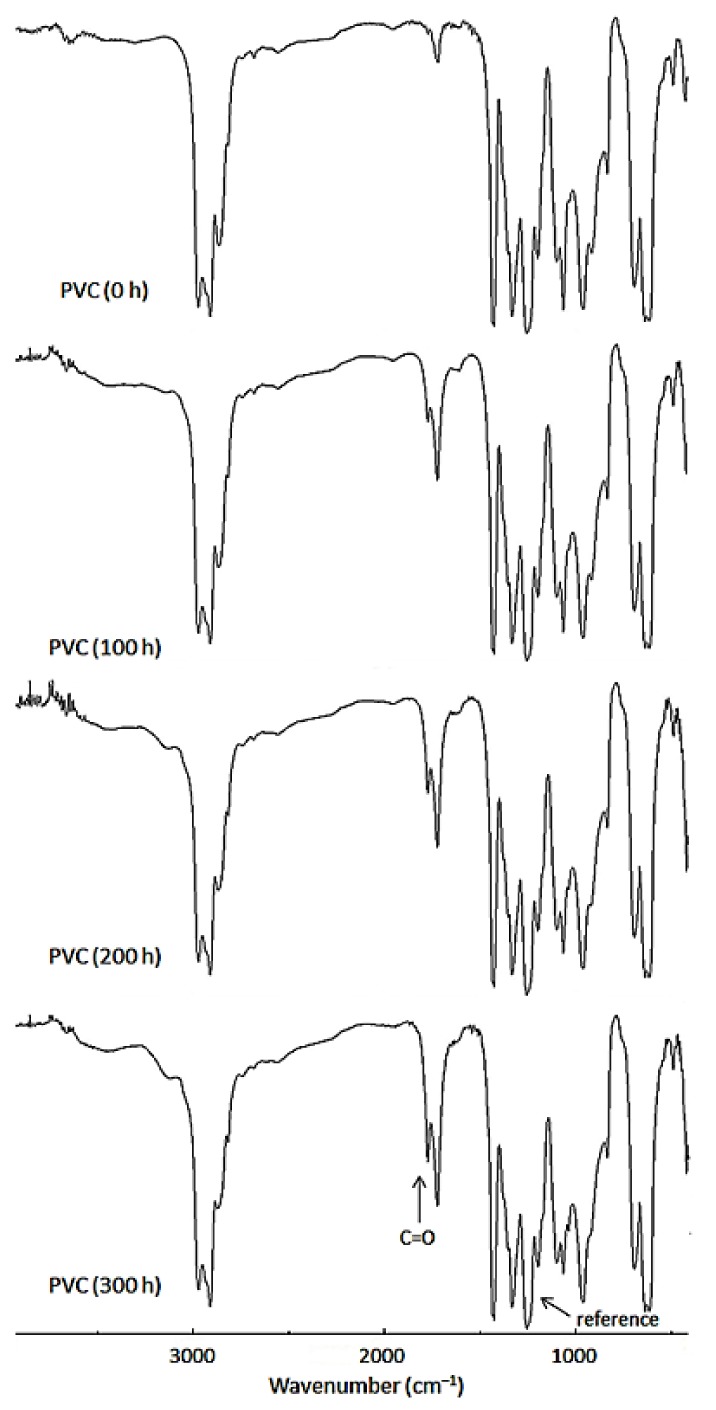
Changes in FTIR spectra of PVC upon irradiation.

Photo-oxidation of PVC leads to several photo-products [41]. The most common oxygenated products are aldehydes, chloroketones, chlorocarboxylic acids, and acid chlorides [41]. The carbonyl-containing fragments can be detected in the FTIR spectra upon irradiation. Therefore, PVC films (40 μm in thickness) were irradiated with a UV light (λ_max_ = 313 nm) for different time periods and the FTIR spectra were subsequently recorded. The FTIR spectra for PVC (blank) before (0 h) and after irradiation (100, 200, and 300 h) are represented in Figure 3.

Figure 3 clearly shows that the intensity of the peak at 1722 cm^–1^—corresponding to carbonyl group (C=O) vibration—became apparent upon irradiation, and its intensity increased as the irradiation time increased. Accordingly, the intensity of the C=O group in the FTIR spectra was monitored upon irradiation and compared to a reference peak of 1328 cm^–1^, which corresponds with the C–H bond in the CH_2_ units of PVC [42]. The carbonyl group index (*I*_C=O_) was then calculated from the absorption intensity for both C=O (*A*s) and reference (*A*r) peaks using Equation (1).

(1) $$I_{s}=A_{s}/A_{r}$$

**Figure 4:**
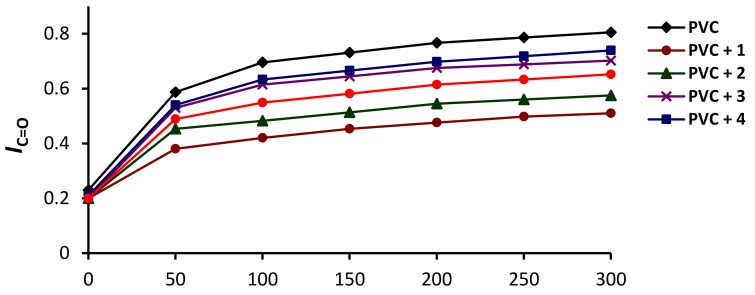
Changes in the *I*_C=O_ of PVC upon irradiation.

The *I*_C=O_ index for the irradiated PVC films, both in the presence and absence of the Schiff bases **1**–**5** (0.5% *w*/*w*), was calculated and plotted against the irradiation time (Figure 4). The changes in the *I*_C=O_ were sharp in the first 50 h and then increased gradually up to 300 h. The changes in the *I*_C=O_ were larger for the PVC (blank) film compared with those obtained for the PVC/**1**–**5** films. For example, the *I*_C=O_ was 0.8 for the PVC (blank) compared to 0.5 for the PVC/**1** blend, after 300 h of irradiation. Evidently, the addition of **1**–**5** led to a significant photostabilization for PVC, with Schiff base **1** deemed to be the most effective additive out of those that were used.

Irradiation of PVC leads to the production of excited electrons that can cause damage to the polymeric chains [43,44]. Azomethine groups within Schiff bases **1**–**5** lead to the release of irradiation energy at a rate that is not harmful to the polymeric chains. The triazine moiety within bases **1**–**5** could also potentially reduce the photodegradation of PVC through the direct absorption of UV light, and as a result, such energy is converted to harmless heat. However, this is less significant when compared with the stabilizing effect of the azomethine group [15]. The polarity of the C=N bond within the triazine rings and azomethine groups could lead to an attraction developing between the nitrogen atoms and the polarized carbons of the C–Cl bonds within the polymeric chains through coordination bonds [15]. Such coordination bonds might help in the transmission of irradiation energy to additives from the excited PVC chains. In addition, bases **1**–**5** act as radical scavengers since they are aromatic rich. Moreover, in the presence of a radical residue (chromophore), the additives **1**–**5** form complexes with PVC chains that can be stabilized through a resonance effect within the aryl rings [45].

**Figure 5:**
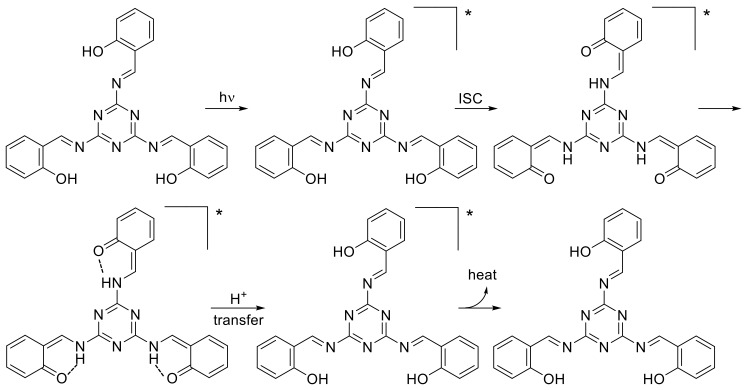
Photostabilization of PVC containing Schiff base **1**. * represents the excited state.

The order of additives in the PVC photostabilization was **1**, **2**, **3**, **5** and **4**. Schiff bases **1**–**3** contain a hydroxyl group, whilst **4** contains a nitro group, and **5** contains a conjugated double bond with the aryl ring. Schiff bases containing hydroxyl groups showed the most stabilizing effect for PVC films compared to the other ones. The hydroxyl group can act as a radical scavenger in which the *ortho*-hydroxy moiety (Schiff base **1**) showed the highest efficiency compared to the others (i.e., Schiff bases **1** and **2**). Such ortho-arrangement leads to the formation of a stable excited state due to hydrogen bonding (Figure 5) as a result of proton transfer and intersystem crossing (ISC) processes between the excited states [46].

### 2.4. Weight Loss

**Figure 6:**
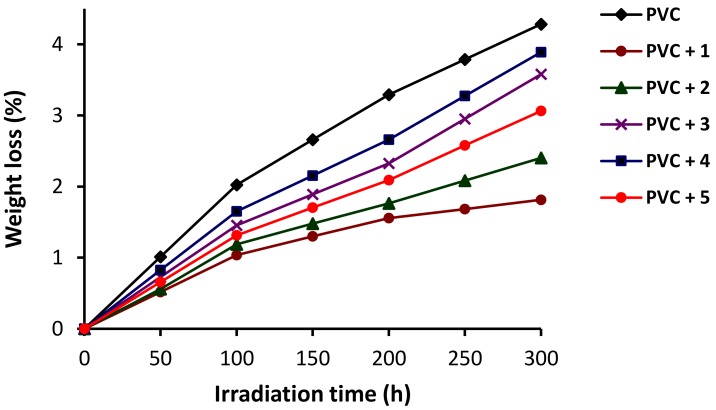
Changes in weight loss (%) of PVC upon irradiation.

The photodegradation or thermal decomposition of PVC leads to the formation of hydrogen chloride and polyene fragments, which cause pyrolysis at high temperature [44]. The PVC dehydrochlorination process leads to a loss in weight along with discoloration and the production of toxic volatile pollutants [47,48]. Therefore, the weight loss percentage due to the irradiation of PVC could be used as a measure for the level of photodegradation. Therefore, PVC films were irradiated, and the weight loss percentage was calculated by subtracting the weight of the film after irradiation (*W*_2_) from the weight before irradiation (*W*_1_) using Equation (2). The changes in weight upon irradiation of the PVC films are shown in Figure 6.

(2) $$Weight\;loss\;\%\;=\;\left[\left(W_{1}-W_{2}\right)/W_{1}\right]\;\times \;100$$

The weight loss of PVC was sharp in the first 100 h of irradiation and was more noticeable in the PVC (blank) film. After 300 h of irradiation, the PVC film in the absence of the Schiff bases lost 4.3% of its weight compared to 1.8% when Schiff base **1** was used. Other Schiff bases led to a weight loss in the range of 2.4–3.9% (after 300 h).

### 2.5. Viscosity Average Molecular Weight

Viscometry provides important information about the shape, dimensions, and size of polymeric particles. The measurement of intrinsic viscosity ([η]) of polymers in solution is a simple and fast method that can be used to determine the average molecular weight (${\bar{M}}_{V}$). The ${\bar{M}}_{V}$ can be calculated using Equation (3), where, *K* and *α* are constants [49,50]. The photodegradation of PVC leads to small molecular weight fragments, which causes a depression in the ${\bar{M}}_{V}$ [51,52].

(3) $$\left[\eta \right]=K{\bar{M}}_{V}^{\alpha }$$

**Figure 7:**
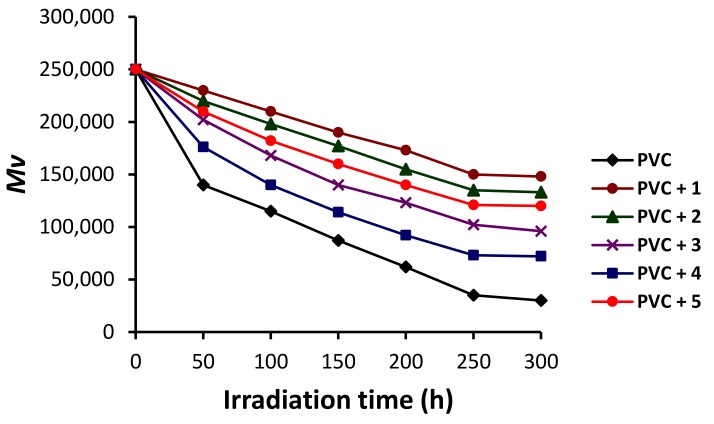
Changes in the ${\bar{M}}_{V}$ of PVC upon irradiation.

Therefore, the PVC films were irradiated, then dissolved in tetrahydrofuran, and [η] was measured after 50 h intervals of irradiation. The changes in ${\bar{M}}_{V}$ (Figure 7) were very sharp at the start of the irradiation process (during the first 50 h) and more or less constant in the last 50 h (from 250 to 300 h). The reduction in ${\bar{M}}_{V}$ was significant in the absence of the Schiff bases compared to those obtained when additives **1**–**5** were used. The ${\bar{M}}_{V}$ of the PVC (blank) dropped from 250,000 to 140,000 after 50 h of irradiation. After 250 h of irradiation, the blank PVC lost 86% of its ${\bar{M}}_{V}$ compared to 40% in the case of the PVC/**1** film.

In order to prove the results shown in Figure 7, the PVC chain sessions (*S*) were calculated using the molecular weight of the non-irradiated PVC films (${\bar{M}}_{V,O\;}$) and those after a specific time (*t*) of irradiation (${\bar{M}}_{V,t}$) using Equation (4). The value of *S* gives an indication of the degree of cross-linking and branching that took place within the PVC polymeric chains due to irradiation [53].

(4) $$S={\bar{M}}_{V,O\;}/{\bar{M}}_{V,t}\;–1$$

**Figure 8:**
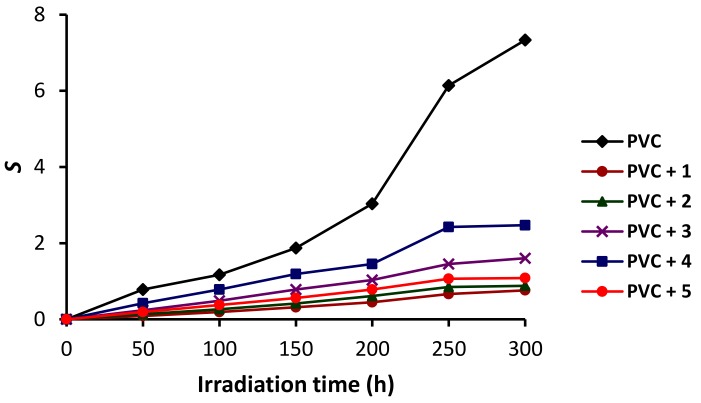
Changes in the *S* values of PVC upon irradiation.

Indeed, the irradiation of PVC films leads to the presence of some insoluble residues forming in tetrahydrofuran as a result of chain sessions. Figure 8 shows that the *S* value was high (7.33) for the PVC (blank) compared with those obtained for the PVC/**1**–**5** films (0.76 to 2.47). Again, the PVC/**1** film produced the lowest *S* value (0.76).

The photodegradation of polymers is also related to the deterioration degree (α), which reflects the number of weak bonds broken in the early stages of the irradiation process. The value of α depends on the molecular weight (*m*), *S* value, and ${\bar{M}}_{V}$ as shown in Equation (5).

(5) $$\alpha =m\times S/{\bar{M}}_{V}$$

**Figure 9:**
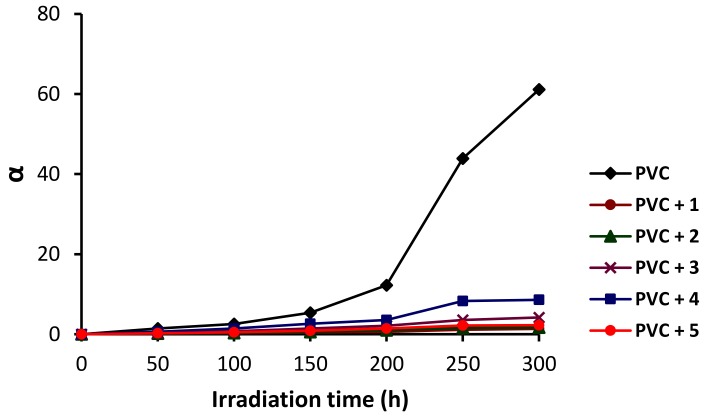
Changes in the α values of PVC upon irradiation.

Therefore, the deterioration degree (α) was calculated for the PVC films and plotted versus the irradiation time (Figure 9). The changes in α value were dramatic for the PVC (blank) film, where α changed from 0 at the start of the irradiation process to 61 after 300 h of irradiation. However, α was very low (up to 8.6) for the PVC/**1**–**5** blends after irradiation (300 h). For the PVC/**1** blend, after 300 h of irradiation, α was also very low (1.3), which clearly highlights the significant and efficient role that Schiff base **1** played as an inhibitor for PVC photodegradation.

### 2.6. Microscopic Surface Morphology

**Figure 10:**
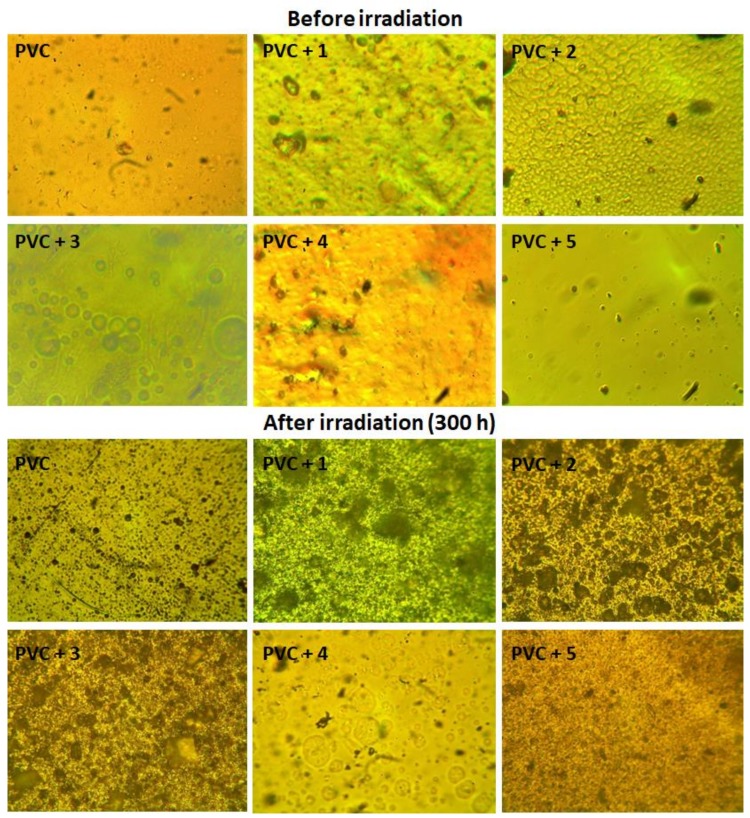
Microscope images (400× magnification) of PVC.

In order to see what effect irradiation could have on the surface morphology of PVC films, a microscope with 400× magnification power was used to inspect the surface. The surfaces of both irradiated and non-irradiated PVC films were inspected, with the captured images being shown in Figure 10. It was clear that the PVC surfaces displayed a high degree of regularity and smoothness before the irradiation process compared with those obtained after irradiation (300 h). The number of cracks and white spots was also significant in the case of the PVC (blank) film when compared with the ones for the PVC/**1**–**5** blends. Such results proved once more that Schiff bases **1**–**5** act as UV absorbers and can reduce the surface damage, photo-oxidation, decomposition, and cross-linking of PVC upon irradiation. The surface morphology of the PVC/**1** blend was less rough, however, and contained fewer numbers of white spots and cracks compared with the surfaces of the PVC containing other Schiff bases.

### 2.7. Scanning Electron Microscopy

**Figure 11:**
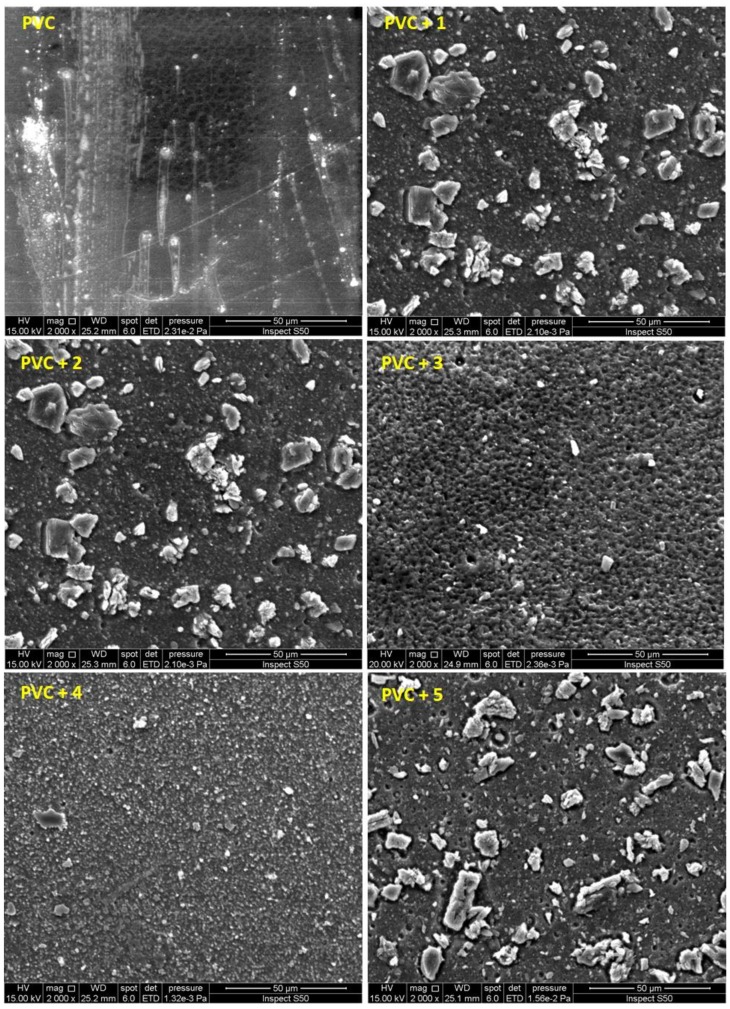
Scanning electron microscopy (SEM) images (50 μm) of PVC after irradiation.

Scanning electron microscopy (SEM) is a useful technique that can be used to test the compatibility of various components within polymeric materials. Such a technique can detect the various interfaces and separation phases within the polymeric matrix, which reflect both mechanical and thermal stability properties, and ionic conductivity [54]. Moreover, SEM images provide information about particle shape and size. It has been reported that the SEM images for the non-irradiated PVC (blank) and PVC/additive blends showed smooth and neat surfaces with a high degree of homogeneity [14,32,33]. The SEM images, captured at an accelerating voltage of 15.00 KV, for the irradiated PVC (after 300 h of irradiation) are shown in Figure 11. The PVC (blank) surface had noticeable damage compared to the damage seen in the PVC films containing bases **1**–**5**. The cross-linking and evolution of hydrogen chloride and other volatile products leads to the formation of an ice-rock-like structure [55,56,57]. Incidentally, the SEM image for the irradiated PVC/**1** film showed the least surface damage.

### 2.8. Atomic Force Microscopy

**Figure 12:**
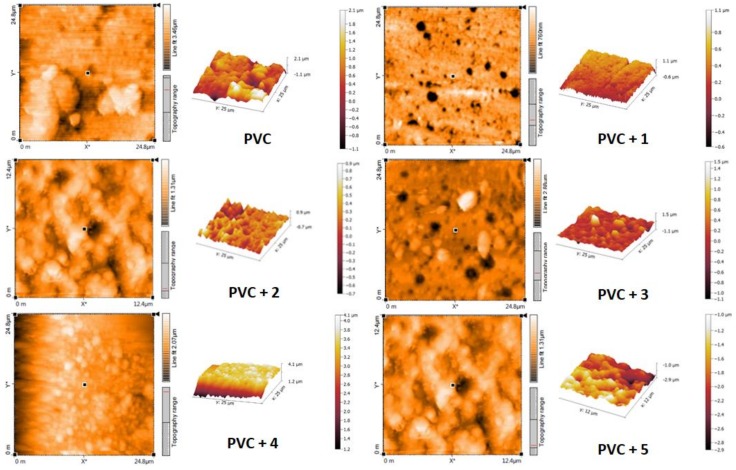
Atomic force microscopy (AFM) images of PVC after irradiation.

Atomic force microscopy (AFM) is a powerful tool used to investigate surface roughness and pore size, and provides two and three-dimensional images for polymer surfaces [58]. Irradiated PVC films (5 × 5 μm^2^) were scanned with AFM and the topographic images produced are shown in Figure 12. Moreover, the AFM images of the PVC films containing Schiff bases **1**–**5** showed spherical shape aggregates (clusters) and fewer holes compared with the blank PVC film.

The surface roughness factor (*R*q) of the irradiated PVC was calculated, the results of which are reported in Table 4. The roughness factor was very high (*R*q = 159.5) for the blank PVC film compared with those obtained for the irradiated PVC/**1**–**5** films (*R*q = 15.5–29.1). The high *R*q for the blank PVC film could be caused by the high rate of dehydrochlorination, which in turn leads to the removal of leachable residues from the surface of the polymer. Evidently, Schiff bases **1**–**5** play a significant role in the inhibition of PVC photodegradation upon irradiation.

## 3. Experimental

### 3.1. General

The PVC powder used in this experiment (grade K67) was provided free of charge from Petkim Petrokimya (Istanbul, Turkey). The melamine, aromatic aldehydes, and the solvents that were used were all obtained from Sigma-Aldrich Chemical Company (Gillingham, UK). The thickness of the PVC film (*ca*. 40 µm) was measured using a Digital Caliper DIN 862 micrometer (Vogel GmbH, Kevelaer, Germany). The PVC morphological images were captured using a Meiji Techno microscope (Tokyo, Japan). The AFM images were recorded on a Veeco instrument (Plainview, NY, USA). The SEM images (accelerating voltage = 15 KV) were captured on an Inspect S50 microscope (FEI Company, Czechia, Czech Republic). A Varian Mercury-300 spectrometer (Varian, Mundelein, IL, USA) was used to record the ^1^H-NMR spectra (300 MHz). The PVC viscosity was measured using an Ostwald U-Tube viscometer (Ambala, Haryana, India). Aluminum plates with a thickness of 0.6 mm (Q-panel Company, Homestead, FL, USA) were used to fix the films. A Kerry PUL 55 ultrasonic bath (Kerry Ultrasonics Ltd., Hitchin, UK) was used for the preparation of the PVC blends.

### 3.2. Synthesis of Schiff Bases ***1***–***5***

Melamine (0.5 g, 4.0 mmol) in dimethylformamide (40 mL) was stirred at 120 °C until the solid completely dissolved. A solution of aromatic aldehyde (13.2 mmol) in dimethylformamide (5 mL) containing glacial acetic acid (0.2 mL) was then added, and the mixture was stirred for a further 6 h under reflux. The mixture was allowed to cool to room temperature, and toluene (120 mL) was added slowly in order to precipitate the products. The solid obtained was collected by filtration, washed with a mixture of methanol and toluene (1:1 by volume), and then dried in a vacuum oven.

### 3.3. Preparation of PVC Films

A solution of PVC (5.0 g) and **1**–**5** (25 mg) in tetrahydrofuran (100 mL) was left in an ultrasonic bath for 30 min. The homogenous solution obtained was cast onto glass plates containing 15 holes (4 × 4 cm^2^; ca. 40 µm), and the films were left to dry at 25 °C for 24 h.

### 3.4. Accelerated Ultraviolet Weathering Tests

The PVC films were irradiated at room temperature using an accelerated weather-meter QUV tester (Philips, Saarbrücken, Germany) equipped with UV-B 313 lamps (λ_max_ = 313 nm; light intensity = 6.2 × 10^–9^ ein dm^–3^ s^–1^).

## 4. Conclusions

Five Schiff bases containing melamine were utilized as photostabilizers for poly(vinyl chloride) films against irradiation with ultraviolet light. The photostabilization of poly(vinyl chloride) films containing Schiff bases was found to have increased significantly. Notably, the polymeric weight loss, reduction in the average molecular weight, and carbonyl group index were low in the presence of the additives when compared to the blank film. The atomic force microscopy, scanning electron micrograph, and the optical microscope images for the poly(vinyl chloride) films containing the additives showed a much smoother surface and a lower number of cracks compared to the blank film after irradiation. In addition, the scanning electron micrograph of the polymer doped with additives looked like a piece of ice-rock in structure—with heterogeneous surface morphology—after undergoing a long period of irradiation.

## Figures and Tables

**Table 1:** Elemental analyses and some physical properties for Schiff bases **1**−**5**.

Additive	Ar	Yield (%)	Melting Point (°C)	Calcd. (Found; %)		
				C	H	N
**1**	2-HOC_6_H_4_	71	293−295	65.75 (66.03)	4.14 (4.34)	19.17 (19.03)
**2**	3-HOC_6_H_4_	78	288−289	65.75 (65.94)	4.14 (4.31)	19.17 (19.22)
**3**	4-HOC_6_H_4_	73	274−275	65.75 (65.95)	4.14 (4.24)	19.17 (19.19)
**4**	4-NO_2_C_6_H_4_	76	266−267	54.86 (54.89)	2.88 (2.97)	23.99 (24.07)
**5**	C_6_H_5_CH=CH	70	274−275	76.90 (77.13)	5.16 (5.91)	17.94 (17.79)

**Table 2:** Some Fourier transform infrared (FTIR) spectral data for Schiff bases **1**–**5**.

Stabilizer	FR-IR (υ, cm^−1^)			
	OH	CH=N	C=N (Ar)	C=C (Ar)
**1**	3332	1632	1539	1465
**2**	3338	1639	1546	1462
**3**	3335	1643	1543	1438
**4**	-	1639	1543	1458
**5**	-	1647	1543	1465

**Table 3:** ^1^H-NMR spectral data for **1**–**5**.

Stabilizer	^1^H-NMR (400 MHz: DMSO-*d_6_*, ppm)
**1**	11.22 (br. s, exch., 3 H, OH), 8.49 (s, 3 H, CH=N), 7.61 (d, *J* = 8.2 Hz, 3 H, Ar), 7.01 (t, *J* = 8.2 Hz, 3 H, Ar), 6.58 (d, *J* = 8.2 Hz, 3 H, Ar), 5.99 (s, 3 H, Ar)
**2**	11.15 (br. s, exch., 3 H, OH), 9.29 (s, 3 H, CH=N), 7.45–7.33 (m, 9 H, Ar), 7.15 (d, *J* = 8.4 Hz, 3 H, Ar)
**3**	10.36 (br. s, exch., 3 H, OH), 9.26 (s, 3 H, CH=N), 7.75 (d, *J* = 8.2 Hz, 6 H, Ar), 6.90 (d, *J* = 8.2 Hz, 6 H, Ar)
**4**	9.28 (s, 3 H, CH=N), 8.44–8.14 (m, 12 H, Ar)
**5**	9.28 (s, 3 H, CH=N), 7.95–7.55 (m, 9 H, Ar), 7.49–7.42 (m, 9 H, CH and Ar), 6.68 (d, *J* = 8.0 Hz, 3 H, CH)

**Table 4:** The *R*q values for the PVC films after irradiation.

Irradiated PVC (300 h)	*R*q
PVC (blank)	159.5
PVC + **1**	15.5
PVC + **2**	24.3
PVC + **3**	27.5
PVC + **4**	29.1
PVC + **5**	26.5
